# Long-term outcomes of post-acute sequelae of SARS-CoV-2 infection: a cohort study protocol

**DOI:** 10.3389/fpubh.2025.1533315

**Published:** 2025-03-07

**Authors:** Dongquan Zhang, Maolin Tong, Xingwen Dong, Chutian Zhang, Yuan Yuan, Xiaojun Wang, Jing Gao, Longfei Guo

**Affiliations:** ^1^Department of Critical Care Medicine, Gansu Provincial Hospital, Lanzhou, China; ^2^The First Clinical Medical College, Gansu University of Chinese Medicine, Lanzhou, China; ^3^College of Natural Resources and Environment, Northwest A&F University, Yangling, China; ^4^Department of Respiratory Medicine, Gansu Provincial Hospital, Lanzhou, China; ^5^Department of Medicine Solna, Karolinska Institutet and Center for Molecular Medicine, Karolinska University Hospital, Stockholm, Sweden; ^6^Department of Respiratory Medicine, University of Helsinki, Helsinki, Finland

**Keywords:** Post-Acute Sequelae of SARS-CoV-2 Infection (PASC), environmental factors, socioeconomic factors, COVID-19, age, long-term health outcomes

## Abstract

**Introduction:**

Post-Acute Sequelae of SARS-CoV-2 Infection (PASC) presents a multifaceted interplay of demographic, clinical, environmental, and socioeconomic factors. Quantification at the individual level of these factors remains underexplored. Our study aims to address this knowledge gap by analyzing the long-term health implications of PASC, utilizing a comprehensive integration of spatiotemporal, clinical, environmental, and socioeconomic data.

**Methods and analysis:**

The study will enroll over 4,000 confirmed COVID-19 patients from Gansu Provincial Hospital, treated from December 2022 to May 2023, as the baseline. These patients are spread across 14 cities in Gansu Province, with geographic coordinates ranging from 92°13′E to 108°46′E and 32°31’N to 42°57’N. Follow-ups will be conducted via structured telephone interviews at 24, 36, and 48 months post-discharge, from 2024 to 2027, to assess PASC and long-term health outcomes. Participants will be categorized into three age groups: children and teenagers (birth to 18 years), adults (18–65 years), and the older adult (over 65 years). Environmental and socioeconomic data corresponding to each case are also integrated. The primary objective is to assess the persistence and long-term health outcomes of PASC symptoms. Secondary objectives focus on evaluating the acute infection phase, its progression, and the efficacy of medical management strategies in influencing PASC trajectories. Mixed-effects models will be utilized to evaluate the impact of various factors on PASC, while spatiotemporal analyses will explore the correlations between environmental and socioeconomic conditions and the diagnosis and recovery trajectories of PASC.

**Ethics and dissemination:**

The Gansu Provincial Hospital’s research ethics committee has approved this study protocol. Participation will be voluntary, with informed consent obtained from all participants. Study results will be published in peer-reviewed journals.

**Clinical trial registration:**

ChiCTR2400091805.

## Introduction

Post-Acute Sequelae of SARS-CoV-2 Infection (PASC), commonly referred to as Long COVID, encompasses a broad spectrum of persistent symptoms and medical complications that continue well beyond the initial COVID-19 infection phase (1–6). Symptoms varies widely, ranging from mild, non-specific issues like fatigue and headaches to severe, debilitating conditions including cardiopulmonary abnormalities and significant neurological deficits (7, 8). These manifestations significantly impair quality of life and pose ongoing challenges to global healthcare systems (9).

The risk factors contributing to PASC are not well understood. A complex array of demographic, clinical, environmental, and socioeconomic elements, may influence PASC (10, 11). There is substantial evidence suggesting that the interplay between biological and environmental components, including air quality, temperature, humidity, and pollution levels, significantly impacts respiratory illnesses, including COVID-19 (12–16). These conditions exacerbate respiratory symptoms and increase the risk of severe complications (14, 15). Socioeconomic disparities also play a crucial role, influencing individuals’ vulnerability and resilience to COVID-19 and its prolonged effects (17). Factors such as income inequality, access to healthcare, housing quality, and employment status profoundly affect an individual’s risk of virus exposure and their ability to receive timely and adequate medical care (18). Education level, social support networks, and community resources are pivotal in determining an individual’s capacity to manage and recover from the infection (19).

Despite established correlations between these variables and outcomes in respiratory and viral diseases, the specific impacts of environmental and socioeconomic conditions on the severity and manifestation of PASC remain insufficiently explored (20–22). Furthermore, the interactions between individual characteristics and environmental risks introduce complex pathways through which PASC develops and progresses (23). For example, residents of socioeconomically disadvantaged areas may encounter increased exposure to environmental hazards and have restricted access to healthcare services, thereby intensifying their risk of developing severe PASC symptoms. Nevertheless, there is a significant gap in comprehensively quantifying these risk factors at the level of individual patients, especially in identifying the interactions between personal characteristics and environmental risks. This deficiency underscores the necessity for an integrated approach that accounts for both individual and environmental risks in the personalized management of PASC patients. Developing a better understanding of these interactions is essential for formulating holistic strategies to prevent and mitigate the long-term health effects of COVID-19 across diverse populations. Such strategies will necessitate enhancing the precision and efficacy of interventions tailored to meet the unique needs of individual patients.

**Figure 1:**
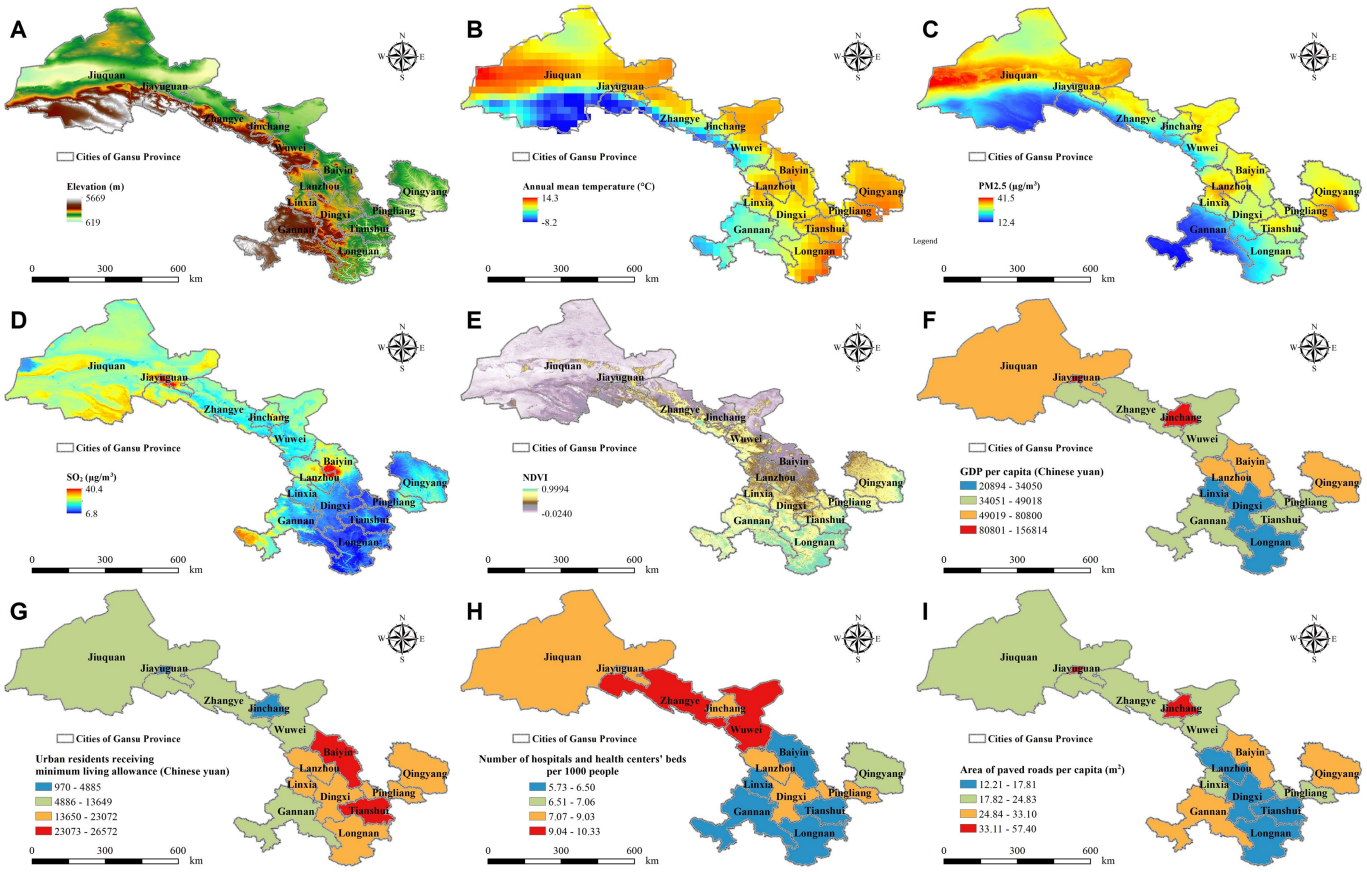
Geographic distribution of environmental and socioeconomic factors across 14 cities in Gansu Province, 2022. This figure illustrates key environmental and socioeconomic indicators across the study region, including: **(A)** Elevation, **(B)** Annual Mean Temperature, **(C)** PM2.5 Concentration, **(D)** Sulfur Dioxide (SO₂) Levels, **(E)** Normalized Difference Vegetation Index (NDVI), **(F)** Gross Domestic Product (GDP) per Capita, **(G)** Median Income of Urban Residents Receiving Minimum Living Allowance, **(H)** Number of Hospital and Health Center Beds per 1,000 People, and **(I)** Paved Road Area per Capita.

Given the complexities inherent in PASC, our research adopts a comprehensive and methodical approach to elucidate how environmental and socioeconomic variables influence the progression and recovery patterns of PASC across diverse demographic segments. This longitudinal study encompasses over 4,000 hospitalized COVID-19 patients from 14 cities across Gansu Province (425,800 square kilometers) (24), leveraging its unique geographic and climatic diversity (Figure 1). Positioned at the intersection of China’s Qinghai-Tibet, Loess, and Inner Mongolia plateaus, Gansu spans a wide range of landscapes, from mountainous terrains to vast plateaus, with elevations ranging from approximately 600 to over 5,600 meters and mean annual temperatures varying between −8 and 14°C (24–26). These environmental conditions are coupled with significant disparities in air pollution levels, as median annual concentrations of fine particulate matter (PM2.5) range from approximately 12–41 μg/m^3^, while sulfur dioxide (SO_2_) concentrations vary from 7 to 40 μg/m^3^ (27, 28). Such fluctuations in air quality, combined with varying levels of urbanization and green space coverage—quantified by the normalized difference vegetation index (NDVI), which ranges from nearly 0 to 1 (29), are likely to influence respiratory health and long-term COVID-19 outcomes. Economic and healthcare disparities further compound these environmental differences, as per capita gross domestic product (GDP) across the region varies nearly tenfold, from approximately 20,000 to over 150,000 Chinese yuan (30). Access to healthcare resources also differs significantly, with some cities having nearly twice the number of hospital beds per thousand people compared to others (30). This broad spectrum of environmental, economic, and healthcare conditions provides an ideal framework for investigating how these factors collectively shape the long-term impacts of COVID-19, particularly in relation to PASC risk and recovery trajectories. By systematically integrating these variables, our study aims to develop a more comprehensive understanding of PASC and inform targeted interventions that address both individual and population-level health disparities.

The diverse environmental and socioeconomic conditions across the study region provide a unique framework for investigating how these factors interact with age-related biological differences to shape the progression and recovery of PASC. This study employs a longitudinal design to systematically track COVID-19 patients over time, allowing for an in-depth analysis of how environmental exposures, economic disparities, and individual characteristics influence PASC risk and long-term outcomes. By integrating spatiotemporal data with patient health records, we aim to capture the dynamic nature of disease progression across different demographic groups. Furthermore, examining the interplay between environmental pollutants, healthcare accessibility, and socioeconomic determinants will offer a more comprehensive understanding of the broader public health implications of PASC. Through this multidisciplinary approach, our study seeks to inform the development of targeted interventions and personalized treatment strategies, ultimately contributing to improved patient care and more effective public health policies.

## Methods and analysis

### Study population

This prospective cohort study, will enroll over 4000 individuals with RT-PCR-confirmedSARS-CoV-2 at baseline, hospitalized at Gansu Provincial Hospital in China from December 2022 to May 2023 (Gansu PASC dataset). Aligning with methodologies from other PASC research, we will gather comprehensive health data during the patients’ initial hospitalization due to COVID-19 (31). The data collection will include extensive hospital records from their acute COVID-19 admissions, capturing demographic information, clinical data, comorbidities, pre-existing symptoms, and other relevant health details.

The patients were from all 14 cities in Gansu Province (92°13′E-108°46′E, 32°31’N-42°57’N), with various environmental and socioeconomic backgrounds. The municipal geographic coordinates of the 14 cities are detailed as follows: Lanzhou (103°51′E, 36°3’N); Jiayuguan (98°23′E, 39°80’N); Jinchang (102°19′E, 38°52’N); Baiyin (104°14′E, 36°55’N); Tianshui (105°72′E, 34°58’N); Wuwei (102°64′E, 37°93’N); Zhangye (100°45′E, 38°92’N); Pingliang (106°66′E, 35°54’N); Jiuquan (98°49′E, 39°73’N); Qingyang (107°64′E, 35°71’N); Dingxi (104°59′E, 35°61’N); Longnan (104°96′E, 33°37’N); Linxia Hui Autonomous Prefecture (103°21′E, 35°60’N) and Gannan Zang Autonomous Prefecture (102°91′E, 34°98’N). Baseline data will be collected using hospital records, and follow-up assessments will be performed at 24-, 36-, and 48-months post-discharge via structured telephone interviews (Table 1). Exclusion criteria are limited to individuals without available contact details, those who deceased during hospitalization or follow-up, those who declined to participate, and those unreachable for follow-up assessments.

**Table 1:** Evaluation parameters at baseline and follow-up visits.

Evaluation parameter	Baseline°	Follow-Up°° (telephone interviews)		
	(Hospital admission)	Visit 1 (24 m)	Visit 2 (36 m)	Visit 3 (48 m)
Informed consent	x			
Inclusion criteria met	x			
Demographics*	x			
Environmental/socio-economic factors†	x			
Symptoms‡	x	x	x	x
Life styles¶	x	x	x	x
Vaccination information**	x			
Reinfection††		x	x	x
Recovery condition‡‡		x	x	x
Comorbidities¶¶	x			
Laboratory test results***	x			
Treatment†††	x			
Clinical outcomes	x			
Medical management‡‡‡		x	x	x
Assessment Tools¶¶¶				
EQ-5D-5L score (Adults)	x	x	x	x
mMRC score (Adults)	x	x	x	x
GAD-7 score (Adults)	x	x	x	x
PHQ-9 score (Adults)	x	x	x	x
FSS score (Adults)	x	x	x	x
ISI score (Adults)	x	x	x	x
EQ-5D-Y (Children 4–15 years)	x	x	x	x
PROMIS (Children 8–17 years)	x	x	x	x
SDQ score (Children 4–17 years)	x	x	x	x

°Baseline Assessment: Conducted at the time of hospitalization for COVID-19 at Gansu Provincial Hospital from 2022 to 2023.

°°Follow-Up Visits: Conducted via structured telephone interviews at three time points from 2024 to 2027.

Visit 1 (24 months post-discharge): PASC diagnosis.

Visit 2 (36 months post-discharge): Long-term outcome assessment.

Visit 3 (48 months post-discharge): Final long-term outcome assessment.

*Demographics: Includes age, gender, ethnicity, marital status (for adults), residential status during infection, education background, personal finances (for adults), work status (for adults), and BMI. These details are tailored to ensure age-appropriateness, with specific personal and professional information omitted for children.

†Environmental/socio-economic Factors: Encompasses sulfur dioxide (SO_2_), nitrogen dioxide (NO_2_), ozone (O_3_), particulate matter 10 (PM_10_), particulate matter 2.5 (PM_2.5_), Normalized Difference Vegetation Index (NDVI), temperature, precipitation, relative humidity, wind speed, per capita GDP, income levels, employment status, job types, education level, and living condition. This comprehensive data helps examine the environmental contexts affecting health outcomes.

‡Symptoms Monitoring: Tracks a wide array of symptoms across various systems.

Respiratory: Difficulty breathing, cough, chest pains.

Digestive: Abdominal pain, diarrhea, nausea, vomiting, loss of appetite, constipation.

Cardiovascular: Palpitations, tachycardia, bleeding.

Musculoskeletal: Arthralgia, myalgia, impaired functioning and mobility.

Nervous System: Headaches, dizziness, “brain fog,” sensory impairments, insomnia, and other sleep difficulties.

Other: Fever, fatigue, skin rashes, irregular menstrual cycles (in females over 12 years of age), erectile dysfunction (in males).

¶Lifestyle Factors: Includes smoking and drinking status, providing insights into behaviors that may influence disease progression and recovery.

**Vaccination Information: Documents the dose and timing of COVID-19 vaccinations, critical for assessing vaccine efficacy and patient immunity.

††Reinfection Data: Tracks instances of SARS-CoV-2 reinfection, including timing and test results (PCR, antigen, self-reported symptoms), to understand immunity and disease dynamics.

‡‡Recovery Assessment: Includes a single question about recovery perception post-COVID-19, with responses categorized as yes, no, or unsure.

¶¶Comorbidities: Comprehensive data collection on conditions such as hypertension, various cardiovascular and respiratory diseases, diabetes, chronic liver and kidney diseases, metabolic disorders, nervous system diseases, psychiatric conditions, cancers, rheumatism, tuberculosis, and anemia, essential for assessing co-impact on health outcomes.

***Laboratory Test Results: Analysis of blood count, electrolytes, renal and liver functions, inflammatory markers, and coagulation profiles to provide a physiological snapshot of health impacts.

†††Treatment: Detailed record of all treatments administered, including medications, oxygen therapy, mechanical ventilation, ICU care, and other interventions, helping to correlate treatment strategies with outcomes.

‡‡‡Medical management: Information of visiting a doctor for any reason and treatment undertaken during follow-up.

¶¶¶Assessment Tools: Includes the 5-level EQ-5D version (EQ-5D-5L), The Modified Medical Research Council (mMRC) Dyspnea Scale, The Generalized Anxiety Disorder-7 (GAD-7), the Patient Health Questionnaire-9 (PHQ-9), The Fatigue Severity Scale (FSS), The Insomnia Severity Index (ISI), The EuroQol Five-Dimension Youth Questionnaire (EQ-5D-Y) assesses health-related quality of life, The Strengths and Difficulties Questionnaire (SDQ), The Patient-Reported Outcomes Measurement Information System (PROMIS).

### Outcomes

#### Primary outcome

The primary objective of this study is to assess the persistence and long-term health outcomes of PASC symptoms. The diagnosis of PASC will be formally established during the first follow-up visit to ensure accurate identification and differentiation from pre-existing conditions, while long-term health outcomes will be evaluated at the second and third follow-up visits (Table 1). Our diagnostic protocol follows the criteria outlined in the WHO definition (32), and utilizes structured telephone interviews for initial PASC assessments (33, 34). PASC symptoms typically emerge at least 3 months after the initial SARS-CoV-2 infection and persist for a minimum of 2 months. To ensure an accurate diagnosis, symptoms must not have been present in the 180 days before infection (35). The initial follow-up visit, conducted 12 months post-diagnosis, confirms the presence and persistence of PASC symptoms, which are systematically evaluated across multiple organ systems, including respiratory, digestive, cardiovascular, musculoskeletal, and neurological functions, as well as general symptoms such as fatigue and fever. Symptom severity is quantitatively assessed using a four-point Likert scale to provide a structured and standardized evaluation of PASC’s impact on patient well-being (36, 37). To further validate symptom emergence and progression, our follow-up protocol differentiates post-COVID-19 manifestations from pre-existing conditions. Aligned with established research protocols, our multidisciplinary (MDR) team conducts follow-ups to collect comprehensive data on new diagnoses and medical consultations, allowing for the identification of secondary conditions potentially associated with PASC (38).

#### Secondary outcomes

The secondary outcomes of this study focus on assessments of the acute infection phase, its progression, the effectiveness of medical management strategies in shaping the trajectory of PASC (38). These assessments are conducted during the follow-up visits, designed to reassess the persistence or resolution of symptoms and evaluate changes in health status since the acute phase of the infection.

The evaluation includes documenting persisting symptoms and identifying any new clinical conditions diagnosed since the initial COVID-19 infection, as well as reviewing medical management strategies such as the reasons for doctor visits and treatments received during the follow-up period. Analysis objectives includes the persistence and progression of symptoms, with a specific focus on respiratory difficulties, overall quality of life, and mental health issues, including anxiety and depression (39). For pediatric populations, the assessment is tailored to specifically monitor the recovery of symptoms relevant to children and adolescents (40), addressing the unique challenges faced by these age groups in the aftermath of COVID-19.

### Variables and data collection

#### Baseline variables

Individual baseline variables will be collected from the Gansu PASC dataset. We gather comprehensive health data during patients’ initial hospitalization for COVID-19. This baseline information serves as a reference to distinguish between pre-existing conditions and symptoms that may be attributed to PASC (41). These variables include demographic factors such as age, gender, ethnicity, marital status, educational background, employment status, and body mass index (BMI). Comorbidities recorded include hypertension, diabetes, respiratory diseases, liver diseases, and other relevant conditions. The Charlson Comorbidity Index (CCI) was adapted using a modified formula to assess morbidity and mortality risks. Conditions were scored as follows: one point for congestive heart failure, chronic pulmonary disease, diabetes, and other conditions; two points for chronic kidney disease and malignant neoplasms; three points for moderate to severe liver disease; and six points for Acquired Immunodeficiency Syndrome (AIDS) (42). Based on the CCI, comorbidities were categorized as mild (0–1), moderate (2–6), or severe (>6). Baseline laboratory assessments included complete blood counts, electrolyte panels, renal and liver function tests, inflammatory markers, and coagulation profiles. Initial treatment protocols involved various medications and supportive care measures, such as oxygen therapy, mechanical ventilation, and immunoglobulin treatments, with emergency interventions including tracheal intubation and cardiopulmonary resuscitation as required.

#### Follow-up assessments

We will conduct telephone follow-ups to diagnose PASC, employing methodologies consistent with those used in major global studies (43, 44). This approach is designed to enhance patient engagement and follow-up compliance, ensuring the inclusion of a broad demographic across diverse geographic locations. Follow-up visits will take place at three time points between 2024 and 2027: Visit 1 (24 months post-discharge) for PASC diagnosis, Visit 2 (36 months post-discharge) primarily for long-term outcome assessment, and Visit 3 (48 months post-discharge) for the final long-term outcome evaluation. During these follow-ups, we will assess the persistence of symptoms and track overall health status, alongside collecting updated information on demographics, environmental exposures, residential status post-infection, lifestyle changes, vaccination history, and hospitalization duration. The monitoring process will also include tracking reinfection cases, post-discharge mortality, the emergence of new symptoms, and changes in health status compared to the initial COVID-19 onset. Consistent with methodologies applied in similar studies (45, 46), our research incorporates patient consultations and provides recommendations for further medical evaluation based on symptoms reported during telephone interviews. However, direct medical interventions are not conducted as part of this study.

#### Environmental data

Environmental data will be collected to assess short-term exposures, including monthly average meteorological conditions and air pollutant concentrations, matched to each patient based on their location and hospital admission period (47). Meteorological data will be obtained from the ECMWF Reanalysis v5 (ERA5) dataset, which provides monthly averaged measurements of temperature, precipitation, relative humidity, and wind speed at a spatial resolution of 0.25° × 0.25° (approximately 25 km at the equator) (48). Air pollution data will be sourced from the China High-Resolution and High-Quality Near-Surface Air Pollutants (CHAP) Dataset, which provides daily concentrations of PM2.5, PM10, ozone (O₃), nitrogen dioxide (NO₂), sulfur dioxide (SO₂), and carbon monoxide (CO) at a spatial resolution of 1 km (49–52). Green space coverage will be assessed using the NDVI, derived from NASA Moderate Resolution Imaging Spectroradiometer (MODIS) Vegetation Indices. NDVI data will be provided annually at a spatial resolution of 1 km (53).

#### Socioeconomic data

Socioeconomic data including city-level per capita, income levels, employment status, job types, education level, and living conditions will be collected during follow-up visits and sourced from the Gansu Development Yearbook, published by the Gansu Province Bureau of Statistic.

### Assessment tools

To ensure a comprehensive and nuanced evaluation across different age groups, the study employs a range of validated assessment instruments. For more detailed descriptions of these tools, please refer to Supplementary Material 1.

#### For adults

(1) The 5-level EQ-5D version (EQ-5D-5L) (54) is used to evaluate health-related quality of life; (2) The Modified Medical Research Council (mMRC) Dyspnea Scale (55) is employed to assess respiratory symptoms; (3) The Generalized Anxiety Disorder-7 (GAD-7) (56) and the Patient Health Questionnaire-9 (PHQ-9) (57) are used for mental health screening; (4) The Fatigue Severity Scale (FSS) (58) is utilized to measure fatigue levels; and (5) The Insomnia Severity Index (ISI) (59, 60) is applied to assess sleep disturbances.

#### For children and adolescents

(1) The EuroQol Five-Dimension Youth Questionnaire (EQ-5D-Y) (61) assesses health-related quality of life; (2) The Strengths and Difficulties Questionnaire (SDQ) (62) evaluates behavioral and emotional health; and (3) The Patient-Reported Outcomes Measurement Information System (PROMIS) (63) is used to assess physical, emotional, and social health indicators.

### Statistical analysis

A variety of statistical methodologies will be employed to analyze the primary and secondary outcomes in this study. The data analysis begins with descriptive and inferential statistical methods to identify patterns and establish correlations. Continuous data will be presented as means with standard deviations (SD), while categorical variables will be reported as percentages. For comparisons across groups, analysis of variance (ANOVA) will be used for continuous variables, and chi-square tests for categorical variables, both with Bonferroni corrections applied in post-hoc analyses. A significance level of *p* < 0.05 will be consistently applied, with adjustments made for multiple comparisons to enhance the reliability of results. Logistic regression models will be employed to evaluate the impact of multiple factors on the severity of PASC. We will utilize a multivariable logistic regression model to analyze the relationship between various independent variables, including demographic factors, clinical characteristics, and environmental exposures, and the binary outcome of PASC presence or absence. The model will integrate variables selected based on clinical relevance and statistical significance derived from preliminary univariate analyses, ensuring that only factors significantly associated with the outcome are included in the final analysis. Variables for inclusion will be chosen based on their potential impact on PASC, informed by both clinical judgment and empirical evidence from initial analyses. We plan to investigate interaction terms, particularly between key demographic and clinical variables, to explore complex interactions that may affect PASC risk. Model fit will be assessed using the Hosmer-Lemeshow test for goodness-of-fit and Receiver Operating Characteristic curve analysis to evaluate predictive accuracy. We will also employ validation techniques such as split-sample validation or cross-validation to confirm the model’s robustness. Sensitivity analyses will be conducted to examine the stability of our results under various assumptions regarding data missingness and potential measurement errors, ensuring that our findings are reliable and applicable across different scenarios. Additionally, advanced analytical techniques will complement these methods, utilizing R (version 4.3.0) with the ‘gpboost’ and ‘stats’ packages and Python (version 3.10) with ‘sklearn’ and ‘gpboost.’

#### Generalized linear mixed-effects models (GLMM) for risk factor estimation

To estimate the influence of risk factors on PASC, Generalized Linear Mixed-Effects Models (GLMM) will be applied. These models will allow for a quantitative assessment of how various environmental and individual-level risk factors contribute to PASC, while controlling for potential confounders. A random intercept for each city will be included to account for non-measurable inter-city variability. Interaction terms between individual and environmental factors will be constructed, with only those demonstrating statistical significance and theoretical or empirical relevance retained for further analysis.

#### GPBoost models and SHapley additive exPlanations (SHAP) for factor quantification

The GPBoost model, which integrates tree-boosting techniques with Gaussian processes and mixed-effects models, will be used to quantify the impact of various factors on PASC. This model is well-suited for handling complex nonlinearities, discontinuities, and higher-order interactions. Gaussian processes provide the flexibility to model random effects and correlations commonly observed in clinical data. Recursive Feature Elimination (RFE) will be employed to streamline the model by iteratively removing the least significant features, as identified through the GLMM, ensuring focus on the most influential variables. Hyperparameter tuning will be performed using five-fold cross-validation to optimize model settings and improve robustness. The SHAP algorithm will be used to quantify each feature’s contribution to the outcome, providing an interpretable measure of feature importance through mean absolute SHAP values.

#### Causal inference models for risk factors and recovery trajectories

Causal inference models will be used to investigate the complex relationships between risk factors and the development of PASC, as well as the recovery trajectories of affected individuals. These models will aim to identify and quantify causal pathways, offering insights into how various factors influence both the onset and progression of PASC. Variables will be selected based on prior assessments, and longitudinal data will be collected to track changes over time. Directed Acyclic Graphs (DAGs) will be employed to visualize potential causal pathways and identify confounding factors that require control during analysis. Structural equation modeling and propensity score matching will be used to estimate the strength and direction of causal relationships. Sensitivity analyses will ensure the robustness and reliability of the findings.

## Patient and public involvement

Patients and public will not involve in the design, recruitment and conduct of the study.

## Ethics and dissemination

This study adheres to the ethical standards outlined in the Declaration of Helsinki and complies with national and local regulations concerning ethical research practices and patient data protection. The Gansu Provincial Hospital research ethics committee has approved this study protocol (ID: 2024–542), and it is registered as a clinical trial (ChiCTR2400091805). Ethical monitoring and data dissemination will be maintained throughout the study.

Informed consent will be obtained from all participants. For pediatric and geriatric populations, specific considerations include obtaining parental consent for minors and ensuring appropriate consent procedures for seniors, particularly those with cognitive impairments. The study commits to maintaining the confidentiality of all medical information, disclosing it only when required by law and with appropriate consent from involved parties. All clinical research data will be retained for a minimum of 5 years following the completion of the study. Study results will be published in peer-reviewed journals.

## Discussion

This study will employ a longitudinal design to investigate how environmental, socioeconomic, and clinical factors shape PASC outcomes in a demographically diverse population. Comprehensive health data collected during the initial COVID-19 hospitalization help distinguish pre-existing conditions from symptoms emerging as part of PASC. By integrating these clinical records with environmental and socioeconomic variables through mixed-effects models and spatiotemporal analyses, the research provides a comprehensive understanding of how both internal factors (e.g., individual health status, comorbidities) and external factors (e.g., air quality, socioeconomic conditions) influence long-term health outcomes following COVID-19. To ensure a representative sample, the cohort is stratified by age, sex, and initial symptom severity, allowing for a detailed and subgroup-specific analysis of PASC manifestations. This multidimensional approach is essential for uncovering the complex interactions between patient-level susceptibility and broader external influences, ultimately informing targeted strategies to improve the management and mitigation of long-term COVID-19 effects.

Existing literature has widely documented the potential influences of underlying comorbidities, demographic factors, and environmental conditions on PASC outcomes (64). Pre-existing conditions such as cardiovascular disease, diabetes, and chronic respiratory disorders have been shown to exacerbate the severity of PASC symptoms, leading to prolonged recovery and an increased risk of complications (65). Individuals with chronic illnesses are more likely to experience severe acute COVID-19, which may predispose them to long-term sequelae due to sustained systemic inflammation and immune dysregulation (66). For instance, patients with diabetes and obesity have demonstrated heightened susceptibility to persistent inflammatory states post-COVID-19, contributing to ongoing fatigue, dyspnea, and cardiovascular dysfunction (67, 68). By examining how pre-existing health conditions interact with PASC, this study aims to elucidate the mechanisms through which chronic illnesses impact recovery trajectories and to identify potential intervention strategies.

Beyond comorbidities, demographic factors such as gender, education level, and marital status may also influence PASC trajectories (69). Gender differences in immune response and health-seeking behaviors have been linked to variations in symptom persistence and severity, with women more frequently reporting prolonged PASC symptoms, including fatigue, neurological issues, and autonomic dysfunction (70). Additionally, education level and marital status serve as proxies for social support and healthcare access, both of which are critical for managing chronic conditions and facilitating recovery (71). Limited health literacy, often associated with lower education levels, may impact adherence to post-COVID care recommendations, while marital status may influence mental health resilience and access to medical resources (72–74). By incorporating these demographic variables into our analysis, we aim to identify groups with greater susceptibility or resilience to PASC and assess how these interactions affect recovery trajectories.

Environmental pressures, such as air quality, urbanization, and access to green spaces, are increasingly recognized as key determinants of post-COVID-19 health outcomes (15, 20, 75). Air pollution has been linked to increased COVID-19 severity and mortality, and higher exposure to PM2.5 and NO2 has been associated with a higher prevalence of long-term respiratory and cardiovascular symptoms in PASC patients (75). Conversely, access to green spaces has been shown to provide protective benefits, improving mental and respiratory health and facilitating recovery (76). Given the environmental diversity of our study sites, this research will assess how these factors contribute to symptom persistence and recovery, leveraging existing knowledge on environmental determinants of health outcomes to refine analytical approaches and enhance the understanding of PASC progression.

A key strength of this study is its extended follow-up period of up to 48 months post-discharge, allowing for a comprehensive assessment of long-term PASC progression and persistence across different age groups. This prolonged observation provides valuable insights into age-specific recovery patterns, refining predictive models for long-term health outcomes and facilitating the development of targeted interventions. Additionally, the use of Geographic Information Systems (GIS) enhances the study by generating disease distribution maps, which help identify environmental and geographic factors associated with PASC. These insights are not only essential for detecting high-risk areas but also for guiding individualized patient care strategies.

Beyond mapping geographic influences, the integration of environmental and socioeconomic data with clinical outcomes strengthens the understanding of how external factors, such as air pollution, economic disparities, and urbanization, affect the severity and trajectory of PASC. Identifying these factors has direct implications for patient care, enabling healthcare providers to incorporate external influences into personalized treatment plans. Time-series analyses further enhance this approach by tracking temporal trends and evaluating the effects of environmental changes on health outcomes, providing a dynamic perspective on how fluctuating conditions contribute to disease progression. Meanwhile, causal inference models help establish robust causal relationships between environmental, socioeconomic, and clinical variables, ensuring a more rigorous evaluation of COVID-19’s long-term effects on PASC. By combining these analytical techniques, the study offers a multidimensional framework for understanding how environmental and socioeconomic conditions interact with individual health factors, ultimately informing more precise treatment and intervention strategies. This research also carries significant implications for patient care, particularly in identifying specific risk factors that exacerbate PASC in vulnerable populations. By considering both internal and external determinants of health, this study supports a shift toward precision medicine, ensuring that interventions address not only biological vulnerabilities but also environmental and socioeconomic disparities that influence recovery.

Despite the strengths of our study, we acknowledge several limitations and have devised strategies to address them effectively. Firstly, our study is based in Gansu Province, China, involving data from 14 cities, each with unique environmental and socioeconomic characteristics. While this provides a rich dataset within a specific geographic region, it limits broader geographic representation. To address this, we have included multiple sites chosen for their environmental diversity, particularly focusing on urban settings across different regions. This strategic selection helps control environmental factors that could influence health outcomes related to PASC. Secondly, our current study setup does not include direct comparisons with other cities or countries, which restricts our ability to generalize findings across different global environments. We plan to expand our analysis to include such comparisons as comparable data becomes available, allowing for a more comprehensive evaluation of environmental and socioeconomic impacts on PASC rates and health outcomes. Thirdly, the non-random nature of our patient recruitment might introduce selection bias, and the reliance on telephone questionnaires could lead to recall bias, particularly as participants may not accurately remember past symptoms. To mitigate these issues, we use advanced statistical methods to control for potential confounders and perform sensitivity analyses to assess the impact of data attrition. Fourthly, an oversight committee is actively involved in monitoring and evaluating the study’s processes to address any unforeseen issues that arise, thereby ensuring the integrity and validity of our research. Plans for further validation through clinical assessments are in place to enhance the reliability of our findings, particularly to address challenges associated with self-reported data. By implementing these strategies, we aim to refine our understanding of PASC and its diverse impacts across different populations, ensuring that our findings are robust and reflective of the true effects of environmental and socioeconomic factors on health outcomes post-COVID-19.

In conclusion, this research represents a critical advancement in understanding PASC by mapping recovery trajectories and identifying both internal and external factors influencing patient outcomes. The insights gained from this study have the potential to transform precision medicine approaches, allowing for more targeted and effective interventions that address the long-term consequences of COVID-19. Furthermore, these findings will contribute to public health strategies aimed at mitigating the impact of PASC, while also supporting the development of tailored patient care approaches based on individual risk factors.

## Platform and data storage

Data from telephone interviews will be meticulously recorded and stored in a password-protected Microsoft Excel file. All study data will be securely housed on a dedicated server at Specific-Disease Database of Gansu Provincial Hospital, with access strictly limited to authorized members of the research team. Rigorous data management protocols will be enforced to ensure the utmost privacy and security of patient information, adhering to the highest standard of data protection.
