# Supplementary material for: Long-term outcomes of post-acute sequelae of SARS-CoV-2 infection: a cohort study protocol
Source: Front Public Health. 2025 Mar 7;13:1533315. doi: 10.3389/fpubh.2025.1533315 (PMC11925897; doi:10.3389/fpubh.2025.1533315)
Supplement: Supplementary file 1 [file Data_Sheet_1.docx]

Adults Questionnaire

Name： Genders：Male
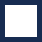
 Female
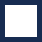
 Your marital status: Unmarried
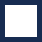
 Married
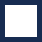
 Your place of residence at the time of your first infection ：Rural
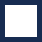
 Urban
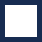


Your educational background: Primary school and below
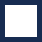
 Junior High School
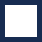


High School
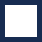
 College and above
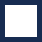


Your monthly income status: No income
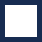
 Below2,000yuan
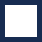
 2000-5000yuan
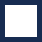
 5000-10000yuan
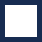
 Over10,000yuan
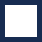


Your work/study status before you contracted: Full-time
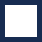
 Part-time
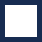
 Unemployed
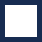
 Partial sick leave
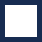
 Full sick leave
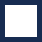
 retirement
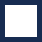


Your current work/study status：Full-time
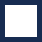
 Part-time
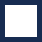
 Unemployed
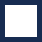
 Partial sick leave
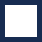
 Full sick leave
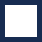
 retirement
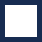


Did you smoke before you got Covid-19?：Yes
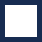
 No
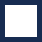
 Do you currently smoke?：Yes
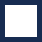
 No
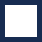


Did you drink alcohol before you got Covid-19?：Yes
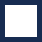
 No
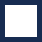


Do you currently drink: Yes
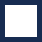
 No
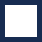


Your height and weight before you got Covid-19： cm/ kg

Your current height and weight： cm/ kg

Your vaccination status：unvaccinated
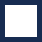
 1dose
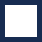
 2doses
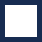
 3doses
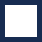


Have you had multiple infections of SARS-CoV-2：No
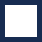
 Yes
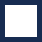


Time of second infection

nucleic acid test
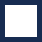
 Rapid antigen tests
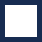
 self-perception
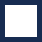


Time of third infection

nucleic acid test
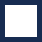
 Rapid antigen tests
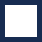
 self-perception
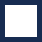


Time of fourth infection

nucleic acid test
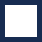
 Rapid antigen tests
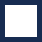
 self-perception
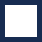


Have you been re-hospitalised for a health problem since you left hospital?

No
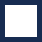
 Yes
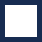
 Time： Y M D Hospital：

**Did you have any co-morbidities prior to infection：**No
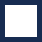
 Yes-name Hypertension
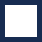
 Cardiovascular disease
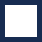
 diabetes：type1
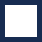
 type2
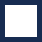
 Chronic obstructive pulmonary disease
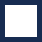
 Asthma
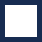
 Pulmonary interstitial fibrosis
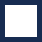
 Sleep apnea syndrome
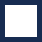
 Bronchiectasis
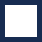
 Chronic liver disease
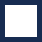
 Chronic kidney disease
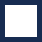
 Metabolic disease
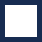
 Nervous system disease
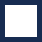
 Psychiatric diseases
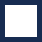
 Malignant neoplasms
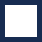
 Rheumatism
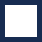
 Tuberculosis
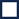
 Anemia
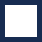


**Presence of newly diagnosed disease after first infection：**No
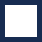
 Yes-name Hypertension
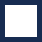
 Cardiovascular disease
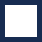
 diabetes：type1
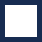
 type2
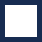
 Chronic obstructive pulmonary disease
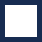
 Asthma
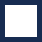
 Pulmonary interstitial fibrosis
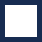
 Sleep apnea syndrome
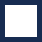
 Bronchiectasis
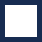
 Chronic liver disease
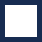
 Chronic kidney disease
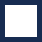
 Metabolic disease
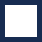
 Nervous system disease
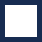
 Psychiatric diseases
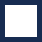
 Malignant neoplasms
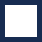
 Rheumatism
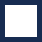
 Tuberculosis
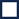
 Anemia
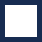


**Any exacerbation of pre-existing conditions：**No
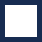
 Yes-name Hypertension
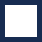
 Cardiovascular disease
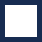
 diabetes：type1
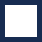
 type2
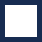
 Chronic obstructive pulmonary disease
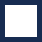
 Asthma
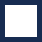
 Pulmonary interstitial fibrosis
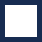
 Sleep apnea syndrome
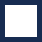
 Bronchiectasis
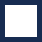
 Chronic liver disease Chronic kidney disease Metabolic disease Nervous system disease Psychiatric diseases Malignant neoplasms Rheumatism Tuberculosis Anemia

Do you feel fully recovered from Covid-19：Yes No Unsure

Do you experience any of the following symptoms? And answer how severe they are.

Current means symptoms lasting three months or more.

**Respiratory**

Difficulty breathing: First infection none mild moderate severe very severe

Current  none mild moderate severe very severe

Cough: First infection none mild moderate severe very severe

Current none mild moderate severe very severe

Chest pains: First infection none mild moderate severe very severe

Current none mild moderate severe very severe

**Digestive**

Abdominal pain: First infection none mild moderate severe very severe

Current none mild moderate severe very severe

Diarrhea: First infection none mild moderate severe very severe

Current none mild moderate severe very severe

Nausea: First infection none mild moderate severe very severe

Current none mild moderate severe very severe

Vomiting: First infection none mild moderate severe very severe

Current none mild moderate severe very severe

Loss of appetite: First infection none mild moderate severe very severe

Current none mild moderate severe very severe

Constipation: First infection none mild moderate severe very severe

Current none mild moderate severe very severe

**Cardiovascular**

Palpitations or tachycardia：

First infection none mild moderate severe very severe

Current none mild moderate severe very severe

bleeding： First infection none mild moderate severe very severe

Current none mild moderate severe very severe

**Musculoskeletal**

Arthralgia： First infection none mild moderate severe very severe

Current none mild moderate severe very severe

myalgia： First infection none mild moderate severe very severe

Current none mild moderate severe very severe

impaired functioning and mobility：

First infection none mild moderate severe very severe

Current none mild moderate severe very severe

**Nervous System**

Headaches： First infection none mild moderate severe very severe

Current none mild moderate severe very severe

dizziness： First infection none mild moderate severe very severe

Current none mild moderate severe very severe

attention deficit or “brain fog”：

First infection none mild moderate severe very severe

Current none mild moderate severe very severe

Loss of taste： First infection none mild moderate severe very severe

Current none mild moderate severe very severe

Loss of smell： First infection none mild moderate severe very severe

Current none mild moderate severe very severe

Insomnia and other sleep difficulties：

First infection none mild moderate severe very severe

Current none mild moderate severe very severe

Fever： First infection none mild moderate severe very severe

Current none mild moderate severe very severe

fatigue： First infection none mild moderate severe very severe

Current none mild moderate severe very severe

skin rashes： First infection none mild moderate severe very severe

Current none mild moderate severe very severe

irregular menstrual cycles（in females over 12 years of age）：

First infection none mild moderate severe very severe

Current none mild moderate severe very severe

erectile dysfunction (in males)：

First infection none mild moderate severe very severe

Current none mild moderate severe very severe

Please describe any other symptoms:

**1.EQ-5D-5L：Under each heading, please tick the ONE box that describes your health TODAY and BEFORE your COVID19 illness**

**MOBILITY TODAY BEFORE**

I have no problems in walking about

I have slight problems in walking about

I have moderate problems in walking about

I have severe problems in walking about

I am unable to walk about

**SELF-CARE**

I have no problems washing or dressing myself

I have slight problems washing or dressing myself

I have moderate problems washing or dressing myself

I have severe problems washing or dressing myself

I am unable to wash or dress myself

**USUAL ACTIVITIES(*e.g. work, study, housework, family or leisure activities*)**

I have no problems doing my usual activities

I have slight problems doing my usual activities

I have moderate problems doing my usual activities

I have severe problems doing my usually activities

I am unable to do my usual activities

**PAIN/DISCOMFORT**

I have no pain or discomfort

I have slight pain or discomfort

I have moderate pain or discomfort

I have severe pain or discomfort

I have extreme pain or discomfort

**ANXIETY/DEPRESSION**

I am not anxious or depressed

I am slightly anxious or depressed

I am moderately anxious or depressed

I am severely anxious or depressed

I am extremely anxious or depressed

We would like to know how good or bad your health is TODAY. Suppose there was a scale numbered from 0 to 100.

100 means the best health you can imagine, 0 means the worst health you can imagine.

Mark an X on the scale to indicate how your health is TODAY. The number =

Mark an X on the scale to indicate how your health was Before your Covid19 illness. The number =

**2.Modified Medical Research Council (MRC) Scale**

0 I only get breathless with strenuous exercise.

1 I get short of breath when hurrying on level ground or walking up a slight hill

2 On level ground, I walk slower than people of the same age because of breathlessness, or have to

stop for breath when walking at my own pace

3 I stop for breath after walking about 100 yards or after a few minutes on level ground

4 I am too breathless to leave the house or I am breathless when dressing.

**3.GAD-7：how often have they been bothered by the following over the past 2 weeks?**

Not at all Several days More than Nearly

half the day every day

1．Feeling nervous, anxious, or on edge 0 1 2 3

2．Not being able to stop or control worrying 0 1 2 3

3．Worrying too much about different things 0 1 2 3

4．Trouble relaxing 0 1 2 3

5．Being so restless that it's hard to sit still 0 1 2 3

6．Becoming easily annoyed or irritable 0 1 2 3

7．Feeling afraid as if something awful might happen 0 1 2 3

Score：

**4.PHQ-9：Over the past 2 weeks, how often haveyou been bothered by any of the following problems?**

Not at all Several days More than Nearly

half the day every day

1．Little interest or pleasure in doing things 0 1 2 3

2．Feeling down, depressed or hopeless 0 1 2 3

3．Trouble falling asleep, staying asleep, or sleeping too much

0 1 2 3

4．Feeling tired or having little energy

0 1 2 3

5．Poor appetite or overeating 0 1 2 36．Feeling bad about yourself -or that you're a failure or have let yourself or your family down 0 1 2 3

7．Trouble concentrating on things, such as reading the newspaper or watching television 0 1 2 3

8．Moving or speaking so slowly that other people could have noticed. Or, the opposite-being so fidgety or restless that you have been moving around a lot more than usual

0 1 2 3

9．Thoughts that you would be better off dead or of hurting yourself in some way 0 1 2 3

Score：

**5.Fatigue Severity Scale**

**Choose a number from 1 to 7 that indicates your degree of agreement with the following statements where 1 indicates strongly disagree and 7 indicates strongly agree. Please answer the questions with reference to how you have beer feeling on average over the last week.**

1.My motivation is lower when l am fatigued

(strongly disagree)1 2 3 4 5 6 7(strongly agree)

2.Exercise brings on my fatigue

(strongly disagree)1 2 3 4 5 6 7(strongly agree)

3.I am easily fatigued

(strongly disagree)1 2 3 4 5 6 7(strongly agree)

4.Fatigue interferes with my physical functioning

(strongly disagree)1 2 3 4 5 6 7(strongly agree)

5.Fatigue causes frequent problems for me

(strongly disagree)1 2 3 4 5 6 7(strongly agree)

6.My fatigue prevents sustained physical functioning

(strongly disagree)1 2 3 4 5 6 7(strongly agree)

7.Fatigue interferes with carrying out certain duties and responsibilities

(strongly disagree)1 2 3 4 5 6 7(strongly agree)

8.Fatigue is among my three most disabling symptoms

(strongly disagree)1 2 3 4 5 6 7(strongly agree)

9.Fatigue interferes with my work, family or social life

(strongly disagree)1 2 3 4 5 6 7(strongly agree)

Score：

**6.Insomnia Severity Index：For each question, please CIRCLE the number that best describes your answer.**

1.Difficulty falling asleep

0None 1Mild 2 Moderate 3 Severe 4Very Severe

2.Difficulty staying asleep

0None 1Mild 2 Moderate 3 Severe 4Very Severe

3.Problems waking up too early

0None 1Mild 2 Moderate 3 Severe 4Very Severe

4.How SATISFIED/DISSATISFIED are you with your CURRENT sleep pattern?

0Very Satisfied 1Satisfied 2Moderately Satisfied 3Dissatisfied 4Very Dissatisfied

5．To what extent do you consider your sleep problem to INTERFERE with your daily functioning (e.g. daytime fatigue, mood, ability to function at work/daily chores, concentration, memory, mood, etc.) CURRENTLY?
0Not at all INTERFERE 1A Little 2Somewhat 3Much 4Very Much INTERFERE

6．How NOTICEABLE to others do you think your sleep problem is in terms of impairing the quality of your life?

0Not at all Noticeable 1A Little 2Somewhat 3Much 4Very Much Noticeable

7．How WORRIED/DISTRESSED are you about your current sleep problem?

0Not at all WORRIED 1A Little 2Somewhat 3Much 4Very Much WORRIED

Score

Children Questionnaire

Name： Genders：Male Female

Your child’s place of residence at the time of your first infection ：Rural Urban

Your child’s educational background: Primary school and below Junior High School

High School College and above Dropout

Did your child smoke before got Covid-19?：Yes No

Does your child currently smoke?：Yes No

Did your child drink alcohol before got Covid-19?：Yes No

Does your child currently drink: Yes No

Your child’s height and weight before you got Covid-19： cm/ kg

Your child’s current height and weight： cm/ kg

Your child’s vaccination status：unvaccinated 1dose 2doses 3doses

Has your child had multiple infections of SARS-CoV-2：No Yes

Time of second infection

nucleic acid test Rapid antigen tests self-perception

Time of third infection

nucleic acid test Rapid antigen tests self-perception

Time of fourth infection

nucleic acid test Rapid antigen tests self-perception

Has your child been re-hospitalised for a health problem since left hospital?

No Yes Time： Y M D Hospital：

**Did your child has any co-morbidities prior to infection：**No Yes-name Hypertension Cardiovascular disease diabetes：type1 type2 Chronic obstructive pulmonary disease Asthma Pulmonary interstitial fibrosis Sleep apnea syndrome Bronchiectasis Chronic liver disease Chronic kidney disease Metabolic disease Nervous system disease Psychiatric diseases Malignant neoplasms Rheumatism Tuberculosis Anemia

**Presence of newly diagnosed disease after first infection：**No Yes-name Hypertension Cardiovascular disease diabetes：type1 type2 Chronic obstructive pulmonary disease Asthma Pulmonary interstitial fibrosis Sleep apnea syndrome Bronchiectasis Chronic liver disease Chronic kidney disease Metabolic disease Nervous system disease Psychiatric diseases Malignant neoplasms Rheumatism Tuberculosis Anemia

**Any exacerbation of pre-existing conditions：**No Yes-name Hypertension Cardiovascular disease diabetes：type1 type2 Chronic obstructive pulmonary disease Asthma Pulmonary interstitial fibrosis Sleep apnea syndrome Bronchiectasis Chronic liver disease Chronic kidney disease Metabolic disease Nervous system disease Psychiatric diseases Malignant neoplasms Rheumatism Tuberculosis Anemia

Does your child feel fully recovered from Covid-19：Yes No Unsure

**Does your child experience any of the following symptoms? And answer how severe they are.**

**Current means symptoms lasting three months or more.**

**Respiratory**

Difficulty breathing: First infection none mild moderate severe very severe

Current none mild moderate severe very severe

Cough: First infection none mild moderate severe very severe

Current none mild moderate severe very severe

Chest pains: First infection none mild moderate severe very severe

Current none mild moderate severe very severe

**Digestive**

Abdominal pain: First infection none mild moderate severe very severe

Current none mild moderate severe very severe

Diarrhea: First infection none mild moderate severe very severe

Current none mild moderate severe very severe

Nausea: First infection none mild moderate severe very severe

Current none mild moderate severe very severe

Vomiting: First infection none mild moderate severe very severe

Current none mild moderate severe very severe

Loss of appetite: First infection none mild moderate severe very severe

Current none mild moderate severe very severe

Constipation: First infection none mild moderate severe very severe

Current none mild moderate severe very severe

**Cardiovascular**

Palpitations or tachycardia：

First infection none mild moderate severe very severe

Current none mild moderate severe very severe

bleeding： First infection none mild moderate severe very severe

Current none mild moderate severe very severe

**Musculoskeletal**

Arthralgia： First infection none mild moderate severe very severe

Current none mild moderate severe very severe

myalgia： First infection none mild moderate severe very severe

Current none mild moderate severe very severe

impaired functioning and mobility：

First infection none mild moderate severe very severe

Current none mild moderate severe very severe

**Nervous System**

Headaches： First infection none mild moderate severe very severe

Current none mild moderate severe very severe

dizziness： First infection none mild moderate severe very severe

Current none mild moderate severe very severe

attention deficit or “brain fog”：

First infection none mild moderate severe very severe

Current none mild moderate severe very severe

Loss of taste： First infection none mild moderate severe very severe

Current none mild moderate severe very severe

Loss of smell： First infection none mild moderate severe very severe

Current none mild moderate severe very severe

Insomnia and other sleep difficulties：

First infection none mild moderate severe very severe

Current none mild moderate severe very severe

Fever： First infection none mild moderate severe very severe

Current none mild moderate severe very severe

fatigue： First infection none mild moderate severe very severe

Current none mild moderate severe very severe

skin rashes： First infection none mild moderate severe very severe

Current none mild moderate severe very severe

irregular menstrual cycles（in females over 12 years of age）：

First infection none mild moderate severe very severe

Current none mild moderate severe very severe

Please describe any other symptoms:

**1.EQ-5D-5L：Under each heading, please tick the ONE box that describes your child’s health TODAY and BEFORE your COVID19 illness**

**Mobility TODAY BEFORE**

I have no problems walking about

I have some problems walking about

I have a lot of problems walking about

**Looking after myself**

I have no problems washing or dressing myself

I have some problems washing or dressing myself

I have a lot of problems washing or dressing myself

**Doing usual activities *(for example, going to school, hobbies, sports, playing, doing things with family or friends)***

I have no problems doing my usual activities

I have some problems doing my usual activities

I have a lot of problems doing my usual activities

**Having pain or discomfort**

I have no pain or discomfort

I have some pain or discomfort

I have a lot of pain or discomfort

**Feeling worried, sad or unhappy**

I am not worried, sad or unhappy

I am a bit worried, sad or unhappy

I am very worried, sad or unhappy

We would like to know how good or bad your health is TODAY. Suppose there was a line numbered from 0 to 100.

100 means the best health you can imagine, 0 means the worst health you can imagine.

Mark an X on the line to indicate how your health is TODAY. The number =

Mark an X on the line to indicate how your health was Before your Covid19 illness. The number =

**2.Strengths and Difficulties Questionnaire**

**For each item, please mark the box for Not True, Somewhat True or Certainly True. It would help us if you answered all items as best you can even if you are not absolutely certain or the item seems daft! Please give your answers on the basis of the child's behaviour over the last six months or this school year**

**Not True Somewhat True Certainly True**

Considerate of other people's feelings

Restless, overactive, cannot stay still for long

Often complains of headaches, stomach-aches or sickness

Shares readily with other children (treats, toys, pencils etc.)

Often has temper tantrums or hot tempers

Rather solitary, tends to play alone

Generally obedient, usually does what adults request

Many worries, often seems worried

Helpful if someone is hurt, upset or feeling ill

Constantly fidgeting or squirming

Has at least one good friend

Often fights with other children or bullies them

Often unhappy, down-hearted or tearful

Generally liked by other children

Easily distracted, concentration wanders

Nervous or clingy in new situations, easily loses confidence

Kind to younger children

Often lies or cheats

Picked on or bullied by other children

Often volunteers to help others (parents, teachers, other children)

Thinks things out before acting

Steals from home, school or elsewhere

Gets on better with adults than with other children

Many fears, easily scared

Sees tasks through to the end, good attention span

**3.Pediatric Fatigue – Short Form 10a**

**Please respond to each question or statement by marking one box per row.**

**In the past 7 days**

Never Almost Sometimes Often Almost

Never Always

Being tired made it hard for me to

keep up with my schoolwork

Being tired made it hard for me to play or

go out with my friends as much as I'd like

I felt weak

I got tired easily

I had trouble finishing things because I was too tired

I had trouble starting things because I was too tired

I was so tired it was hard for me to pay attention

I was too tired to do sports or exercise

I was too tired to do things outside

I was too tired to enjoy the things I like to do

**Consent Form**

We are currently conducting an Long-Term Outcomes of Post-Acute Sequelae of SARS-CoV-2 Infection: A Cohort Study Protocol. The study protocol has been approved by the Gansu Provincial Medical Committee for ethical review and consent to conduct the clinical study. Please read the following as carefully as possible before you decide whether or not to participate in this study.

**Introduction of the research project**

The unit responsible for this study is Gansu Provincial People's Hospital.

**Research target：**the study aims to investigate the effects of environmental and socioeconomic factors on PASC and analyze their impacts on recovery patterns across different age groups.

**Research content：**This study employs a longitudinal design to investigate the complex dynamics between environmental and socioeconomic factors and their impact on the PASC within a demographically varied population. By integrating detailed clinical records with environmental and socioeconomic data through mixed-effects models and spatiotemporal analyses, the research provides a sophisticated examination of the multifaceted influences on health outcomes following COVID-19.

**Type of study:**

Prospective observational study

**Benefits to Subjects of Participating in this Study:**

Participation in this study facilitates close monitoring of your condition, helps to understand your own recovery from neocoronavirus infection, and contributes to early detection of long-term neocoronavirus symptoms and early clinical intervention. There are no costs associated with the study.

**Ethics**

This study strictly complied with the ethical guidelines of the Declaration of Helsinki for medical research on human subjects, respected the personality of the subjects and protected their rights and interests. The study protocol and the Informed Consent Form have been approved by the Medical Ethics Committee of Gansu Province. Even if the results of the study are published, your personal information will not be disclosed. Patient Declaration After the introduction by the doctor, I have read all the contents of the Informed Consent Form in detail, understood the background, method and purpose of this study, obtained complete and true information related to this study, and gave my consent without any pressure and free choice. I fully understand and support this study, and voluntarily participate in this study, and am willing to cooperate with the doctor in charge, give a true and objective medical history, undergo a physical examination, and complete this study.

Patient's signature: Date:

Signature of the doctor: Date:

(Add or replace the following when the subject's ability to give informed consent is lacking or inadequate:)

Signature of legal representative: Date:

Relationship to Subject:

Signature of subject (8 years of age and older): Date:
